# 110 years of rice breeding at LSU: realized genetic gains and future optimization

**DOI:** 10.1007/s00122-025-04913-z

**Published:** 2025-06-09

**Authors:** Allison Vieira da Silva, Adam Famoso, Steve Linscombe, Roberto Fritsche-Neto

**Affiliations:** 1 Department of Genetics, “Luiz de Queiroz” College of Agriculture, University of São Paulo (USP), Piracicaba, São Paulo Brazil; 2 Louisiana State University Agricultural Center, Rice Research Station, Rayne, LA 70503 USA

## Abstract

**Key message:**

Strategic resource allocation in breeding programs is key to balancing cost-effectiveness and genetic improvement.

**Abstract:**

This research aimed to understand the critical role of adopting advanced breeding tools and optimizing breeding strategies to ensure the sustainability and success of public breeding programs in meeting future food security challenges. In this context, there are two main objectives: estimate the genetic gains achieved over 110 years in the rice breeding program of Louisiana State University (LSU); evaluate through stochastic simulations the impacts of modern selection tools such as genomic selection (GS) and high-throughput phenotyping (HTP) on future genetic gains. Considering the 110 years, the average increase was 4.55 kg/ha per generation (23 breeding cycles). However, from 1994 to 2018, we observed more substantial trends in genetic gains, particularly for grain yield, which increased by approximately 56.54 kg/ha per year. Based on simulations, integrating GS and HTP demonstrated significant advantages, including shorter breeding cycles, enhanced selection accuracy, and reduced costs. Also, simulation results showed that this approach yielded the highest response to selection (4.68% per year) due to the synergistic effects of combining advanced phenotyping techniques with GS. Finally, we assessed the effects of balancing the number of parents, crosses, and progeny sizes to maximize genetic gains and maintain genetic variability. Variance component analysis indicated that progeny size had the greatest impact on total variance (36%), followed by the number of crosses (23%) and the number of parents (3.4%). The findings highlight the need for strategic resource allocation in breeding programs to balance cost-effectiveness and genetic improvement.

**Supplementary Information:**

The online version contains supplementary material available at 10.1007/s00122-025-04913-z.

## Introduction

Public plant breeding programs have a great responsibility for global food security, as this sector is often the main source of genetic gain increases for the neediest communities (Cobb et al. 2019). Many of them have long-term integrated activities such as basic research, germplasm improvement, and the development of improved varieties; these are essential activities of a public plant program and cannot be sustained without continuous public funding (Coe et al. 2020). As a result, developing and adopting high-yielding varieties have significantly contributed to increasing agricultural productivity and reducing hunger over the last century. Their contribution will likely continue growing over time (Qaim and Kouser 2013). In this context, genetic gain is one of the main benchmarks of success for a breeding program, and with a high and sustained rate over cycles, genetic gain plays a central role in agricultural transformation (Xu et al. 2017).

There is a projection regarding the main staple crops worldwide. A linear progression of genetic gain, targeting an established rate of 2% per year, must be achieved to overcome the so-called "2050 challenge" and match the world population growth (Li et al. 2018). Breeding programs have a major impact on increasing crop yield at the necessary rate to match the needs of the world's growing population and achieve global food security (Cobb et al. 2019). Estimating genetic gain is one of the strategies to track the performance of a breeding program on this mission (Xu et al. 2017). It can be a useful indicator of the efficiency of the breeding program in utilizing financial and genetic resources sustainably through the breeding process (Covarrubias-Pazaran 2020). This means that the use of resources is effectively reflected in genetic progress toward the breeding program's target goals. In addition to increasing productivity, breeding programs can have different goals, such as disease resistance and tolerance to soil salinity and drought. The breeding program's target goal directly impacts the genetic gain estimates. In this context, factors such as breeding scheme, trait heritability, connectivity, and the number of trials and sites per genotype can also introduce errors in these estimates (Rutkoski 2019a, b; Raymond et al. 2023). Therefore, genetic gain estimates should not be used to compare the efficiency of different breeding programs directly. Instead, they should be employed to assess the presence of a positive upward trend or to obtain a close estimate of the realized genetic gain within a single breeding program (Rutkoski 2019a, b).

Consequently, several studies have assessed the rice genetic gain of other rice breeding programs worldwide for grain yield. Streck et al. (2018) estimated the genetic gain of the irrigated rice breeding program of Embrapa in Southern Brazil (from 1972 to 2016) and observed a genetic gain of 0.62% per year (37.91 kg/ha/year). Kumar et al. (2021) estimated the genetic gain for rice yield in India from 2005 to 2014. They observed a positive genetic trend in grain yield of 0.68% (34 kg/ha/year) under irrigated control, 0.87% (25 kg/ha/year) under moderate drought stress, and 1.9% (27 kg/ha/year) under severe drought stress. Khanna et al. (2022) estimated the genetic gain of the International Rice Research Institute (IRRI) rice drought breeding program, between the years 1980–2015, at the rate of 0.23% (10.22 kg/ha/year) under non-stress conditions and 0.13% under drought conditions (2.29 kg/ha/year). Khanna et al. (2024) estimated the genetic gains in IRRI’s salinity breeding program from 2008 to 2019 and Bangladesh from 2005 to 2014. They observed a positive genetic trend of 0.1% (1.52 kg/ha) per year in IRRI, Philippines, and 0.31% (14.02 kg/ha) in Bangladesh. Also, in IRRI, Juma et al. (2021), for the irrigated rice breeding program, analyzed 102 historical yield trials conducted in the Philippines from 2012 to 2016 and represented 15,286 breeding lines (including released varieties). They estimated a rate of genetic gain for grain yield at 8.75 kg/ha/year (0.23%) for crosses made from 1964 to 2014. Reducing the data to only IRRI-released varieties, the rate doubled to 17.36 kg/ha/year (0.46%). Regressed against the breeding cycle, the rate of gain for grain yield was 185 kg/ha/cycle (4.95%). In turn, Rahman et al. (2023) have estimated a genetic gain of the Bangladesh breeding program from the years 1970–2020, at the annual rates of 0.28% for winter rice and 0.18% for monsoon rice, which corresponds to an increase of 10 kg/ha per year in grain yield for both. More recently, Seck et al. (2023) reviewed 29 rice studies conducted between 1999 and 2023, covering different regions, traits, periods, and estimation methods. The genetic gain for grain yield, in particular, showed significant variation, ranging from 1.5 to 167.6 kg/ha/year, with a mean value of 36.3 kg/ha/year.

Given the numbers above, it is easy to realize that plant breeding is a complex long-term activity, and every decision has to consider the huge amount of time and resources invested in it. Even with the amount of information we have generated in the last decades, adopting new tools in plant breeding demands a lot of caution. The time and resources needed to develop the plant breeding practices do not allow for many tests of new strategies. In addition, comparing breeding strategies based only on field trials could be risky once a unique or a couple of field trials is a random sample and does not represent all possible outcomes of a random effect, leading to low-reliability results (Li et al. 2012). Thus, stochastic simulation can help the breeder overcome those aspects and project the future once the breeders have a cost-effective way to simulate breeding scenarios to optimize a breeding program. Moreover, allows us to test new strategies and tools through many cycles, with many repetitions to estimate random effects better, and all of those with the cost of a powerful computer machine and advanced knowledge in quantitative genetics and plant breeding to simulate relevant scenarios (Bančič et al. 2024).

To accomplish future demands, the adoption of advanced tools such as genomic selection (GS) and high-throughput phenotyping (HTP) is indispensable to improving genetic gain rates (Cobb et al. 2019), of course, when all other components of the breeding program are already optimized (Rutkoski 2019b; Seck et al. 2023). The main advantages of genomic selection (GS) include the reduction of breeding cycle length, higher selection accuracy of top-performing genotypes, and reduced costs associated with classical phenotyping (Crossa et al. 2017). In addition to the costs of manual phenotyping, measurements are prone to human error and subjectivity. HTP tools can increase selection accuracy at lower costs and with higher selection intensity, enabling the phenotyping of more plots (Xu et al. 2021). HTP can be implemented using phenotyping platforms (Yassue et al. 2022) and remote sensing with unmanned aerial vehicles (Hassan et al. 2019). The advantages of GS and HTP may be further enhanced when these tools are combined. The synergistic effect between GS and HTP has been observed in many empirical studies involving crops such as wheat (Sun et al. 2019), maize (Adak et al. 2024), and soybean (Moreira et al. 2021), but it is not so evident in rice yet.

The Louisiana State University (LSU) rice breeding program was established in 1908 and has been instrumental in advancing the rice industry in Louisiana (USA) and globally. Since then, we have released 63 varieties and groundbreaking technologies, such as the Clearfield® Rice (Sudianto et al. 2013). Despite the program's evolution and success over the years, the primary objectives and priorities have remained consistent, focusing on the release of improved varieties. However, to design new strategies for the future, we need to quantify the success of our past efforts and then define the best balance between labor and budget and genetic gains for future initiatives. Thus, the main objectives of this work were to: (i) estimate the genetic gains achieved in 110 years of Louisiana State University's (LSU) long-grain rice breeding program, (ii) use stochastic simulations to evaluate the impacts of adopting modern selection tools, such as GS and HTP, and the effect of balancing the number of parents, crosses, and progeny size on genetic gain and resource allocation.

## Material and methods

### The LSU rice breeding framework

We have used a recurrent selection approach involving multiple rounds of selection and interbreeding among superior offspring for subsequent selection cycles. This program operates through a closed-loop germplasm pipeline, concentrating on population improvement. It employs a tiered trial system wherein top-performing lines progress toward potential varietal release. In the early breeding stages, selections focus on highly heritable traits like plant height, maturity, and type. As the cycle progresses, the emphasis shifts from high heritable qualitative traits to lower heritable traits like grain yield and milling characteristics. Field evaluations for trait assessments are conducted locally at LSU and through multi-environmental trials across the Louisiana rice region. For adaptation trials, evaluations are extended to neighboring states to evaluate stability and adaptability to other production areas. Furthermore, the increase in seed numbers and generational advancements occur in Puerto Rico, contributing to the program's meticulous and comprehensive breeding operations.

The breeding process starts with hybridization and population development, with approximately 300 new populations developed annually. A critical component of the population development stage is choosing the right parents and determining the size and number of populations. The F_1_ generation is grown at LSU, and each population is confirmed by DNA marker testing. The *F*_2_ generation is immediately planted at the winter nursery in Lajas, Puerto Rico. Panicles are selected from single *F*_2_ plants and advanced to the line development stages at the LSU, consisting of approximately 20,000 *F*_2_:_3_ to 9000 *F*_3:4_ panicle rows. The line development stage, lasting 1–2 years, aims to achieve crucial objectives: firstly, ensuring the homogeneity and inbreeding of the lines; secondly, selecting highly heritable traits; and thirdly, increasing seed quantity to provide a sufficient supply for future trials. Moreover, the marker-assisted selection (MAS) strategy is employed at these early stages to aid in selecting qualitative traits.

Subsequently, the selected lines progress to preliminary yield testing (PYT), marking the initial evaluation stage in plots. This phase represents the parents to be selected for the next cycle and is a valuable GS training set (TS) source. The primary objective at this juncture is to identify and select the most promising materials to advance to the subsequent stage. Conducted across two environments, involving two planting dates with one replicate each, this evaluation typically includes *F*_3_:*F*_5_ materials and is approximately 1500 entries across all segments combined.

Finally, regional and advanced yield testing (RYT and AYT, respectively) are conducted across multiple environments and locations. The RYT test is conducted across 4–5 environments. At this stage, we have approximately 200 entries distributed in a randomized complete block design (RCBD) with two replicates, comprising materials advanced from the PYT stage. The AYT is conducted across eight environments with 10 entries and three replicates, also arranged in a randomized complete block design (RCBD), comprising materials advanced from the RYT stage or lines repeated from the previous year’s AYT. The AYT entries are primarily F_5:7_ materials. At these stages, a major emphasis is on evaluating key quantitative traits and assessing the response to the environment and stability. The stage determines the breeding cycle, and we select the parents to compose the next generation.

The most promising lines being considered for potential commercial release undergo a pre-commercial (PC) testing stage. This test is conducted across 25–30 environments in collaboration with the University of Arkansas, Horizon Ag., and Nutrien Ag. Each collaborator nominates five lines to be included in the trial each year. At the PC stage, we have three replicates and it comprehensively tests yield, agronomics, grain quality, and disease resistance. This rigorous evaluation ensures a robust understanding of the potential new varieties before release.

### Estimating the realized genetic gains

With the aid of current and former researchers of the LSU rice breeding program, phenotypic information from the last 24 years (1999–2022) was gathered, as well as the whole pedigree (1615 parents) since the founders (110 years). The data correspond to 26,882 plots, 597 genotypes, and 178 trials in 19 locations in completely randomized blocks. The genotypes to be evaluated will be grain yield, plant height, and whole milling. The traits evaluated presented zero or a low correlation among them (Supplementary Fig. 2). To capture the environmental trend over time, we estimated the yield trend of check genotypes that were present in at least 60% of the years from 1999 to 2022. These checks serve as a consistent benchmark across environments and help to account for non-genetic changes in yield over the years (Supplementary Fig. 3).

The data were processed, organized, and subjected to statistical quality control and descriptive statistical analyses. The distribution of the data and the presence of outliers were checked. The Bonferroni test was used for outlier removal. The Bonferroni *p*-values for testing each observation, in turn, are a mean-shift outlier based on standardized residuals in linear (*t*-tests), generalized linear models (normal tests), and linear mixed models. More details can be found at Fox and Weisberg (2019).

After quality control, the data were modeled in three stages. In the first stage, for each trial and each of the three evaluated traits, a mixed model was used to estimate the adjusted means and weights for each genotype, in each of the trials and for the three traits via REML (restricted maximum likelihood) method using the *sommer* package in the R environment (Covarrubias-Pazaran 2016):

1 $${\mathbf{y}} \, = \, {\mathbf{Xg}} \, + \, {\mathbf{Zr}} + {\mathbf{e}}$$

where **y** is the vector of phenotypic values; **g** is the fixed effect of genotype; **r** is the random effect of replicate, where **N**(0, **I**σ_r_^2^); **e** is the random effect of the residue, where **N**(0, **R**σ_e_^2^); **X** is the incidence matrix for the fixed effect, in this case, genotypes; **Z** is the incidence matrix for the random effect. Also, the same model was adjusted considering genotype as a random effect to estimate the components of variance and the plot basis broad-sense heritability:

2 $$H^{2} = \frac{{\sigma_{G}^{2} }}{{\sigma_{G}^{2} + \sigma_{E}^{2} }}$$

where $\sigma_{G}^{2}$ corresponds to the genetic component of variance and $\sigma_{E}^{2}$ corresponds to the residual component of variance. Heritability was used as a quality control for the trials. All trials that showed heritability lower than 0.35 were removed from further analyses (13 of 434), less than 3%. To find more details, a supplementary file summarizes all trials, traits, heritability, missing data, year, and location (Supplementary File 1).In the second stage, a joint analysis was conducted, considering the following model:

3 $${\mathbf{y}}^{*} = {\mathbf{X}}_{1} {\mathbf{g}} + {\mathbf{X}}_{1} {\mathbf{t}} + {\mathbf{e}}$$

where y* corresponds to the adjusted means obtained in the first step of each line:trait:trial combination. The fixed effect of genotype is represented by **g**; the fixed effect of year is of field evaluation represented by **t**; **e** corresponds to the random effect of the residue with $N(0,\sigma_{{\text{e}}}^{2} {\mathbf{W}})$ (Krause et al. 2023). The matrices **X**_1_ and **X**_2_ are the incidence matrices for the fixed effects of the genotype and the year, respectively. **W** is a diagonal matrix with the weights, and **I** is an identity matrix. This step is important to minimize the year of evaluation effect to estimate more reliable genetic values. For that, the connectivity over the years is vital. Both the first and second steps generated adjusted means, corrected for a set of effects described in both models presented.

**Fig. 1:**
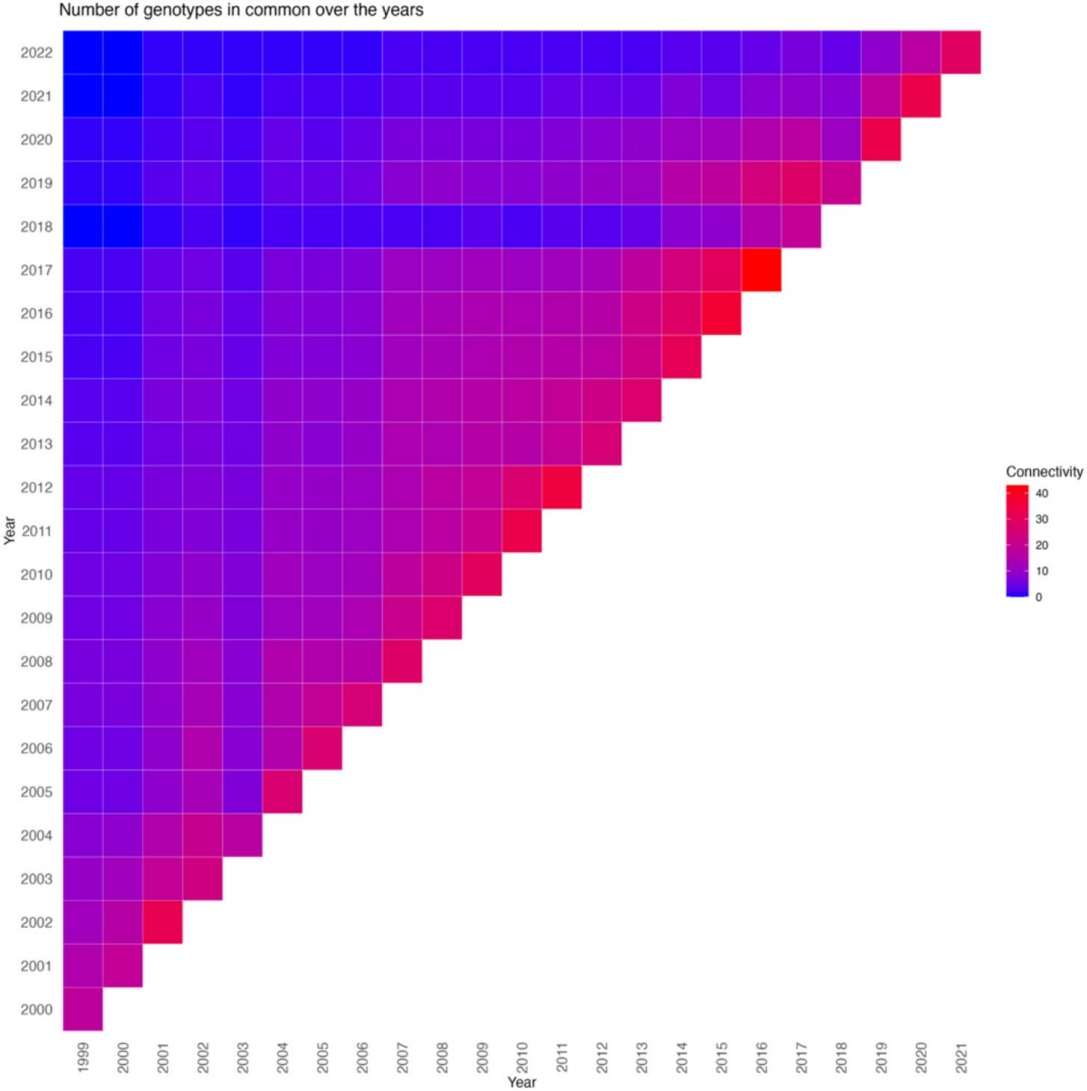
Percentage of genotypes shared between each two-year combination from 1999 to 2022. They ranged from 14 to 36%, with only six of the 24 years showing a percentage lower than 20%. Connectivity corresponds to the number of genotypes shared by year, represented by the colors of the dots ranging from 17 to 43 genotypes

The percentage of shared genotypes across the years ranged from 14 to 36%. Between 2003 and 2004, the shared genotype percentage was 14%, serving as a linkage between the 2 years, while between 2016 and 2017, this figure rose to 36%, indicating a higher degree of continuity between these years. In 24 years evaluated, a connectivity rate below 20% was noted in only six instances. Conversely, the remaining 18 years demonstrated connectivity rates exceeding 20%, highlighting a consistent linkage between the evaluated genotypes and the subsequent year (Fig. 1).

To estimate the breeding value of the founders or early breeding generations (predict the past), a variation of this model was used, including the pedigree matrix (**A**), obtained from the whole historical data, composed of 1615 parents over the 110 years (Supplementary Fig. 1):

4 $${\mathbf{y}}* = {\mathbf{X\beta }} + {\mathbf{Z\alpha }} + {\mathbf{e}}$$

where y* corresponds to the adjusted means for the evaluated trait from the first step of each line:trial combination; **X** corresponds to the incidence matrix of fixed effect for the years of field evaluation; **β** corresponds to the vector of year fixed effects; **Z** corresponds to the incidence matrix of random (genetic) effects; α corresponds to the additive genetic effect, where **α** ~N(0, **A**σ_a_^2^); and **e** corresponds to the random effect of the residue with $N(0,\sigma_{{\text{e}}}^{2} {\mathbf{W}})$, (Krause et al. 2023). As described earlier, **W** is a diagonal matrix with the weights.

Finally, in the third step, a regression for each trait was performed to estimate the genetic gains, following the model:

5 $$y** \, = \, a \, + \, Xi \, + \varepsilon$$

where y** is the vector of adjusted means or breeding values from the second step analysis of the lines for each trait evaluated; ***a*** is the intercept of the regression, proving the overall population performance; ***i*** is the slope of the regression, related to the population improvement over the years (in this case, year is the year the cross was made or the genotype was introduced; in other words, when the genotypes were obtained); ***ε*** is the residue random effect, where ε ∼ *N*(0, *I*$\sigma_{e}^{2}$). The genetic gain was estimated by dividing the slope by the intercept. At this stage, we did not apply the weights, as they would be homogeneous due to the adjustments made in the previous two stages.

Based on the pedigree records, the generations were calculated using the countGen function from the *pedigree* R package, version 1.4.2. Each individual's generation was determined by iterating through the pedigree and assigning a generation number: one plus the highest generation number of the known parents or zero for founders. The generation interval was approximately 5 years, resulting in 23 generations over 110 years.

## Optimizing the future breeding generations

We used the R package *AlphaSimR* (Gaynor et al. 2021) to simulate different breeding strategies and numbers for the stochastic simulations. Each scenario described below was simulated over 20 breeding cycles replicated 100 times and compared regarding population performance improvement over a 15-year horizon.

### Crop history of evolution and genetic parameters

We simulated an initial historical population of 1000 inbred individuals, featuring 1644 segregating loci distributed across 12 chromosomes, averaging 137 loci per chromosome, by the “GENERIC” option and diploid species. Then, for the initial parameters of the target quantitative trait, such as grain yield, we defined the existence of 360 quantitative trait loci (QTL) controlling the trait (30 quantitative trait nucleotide (QTN) per chromosome), an SNP chip with 45 markers per chromosome, totaling 540 high-quality markers. Moreover, SNP and QTN sites were not allowed to overlap. The additive, dominance, and average degree of dominance parameters were defined based on Li et al. (2008). We assigned additive and dominance effects to each QTN. Total genetic values for each genotype were obtained by summing all additive and dominance effects times the appropriately scaled genotype dosage for all QTN; for details, see Gaynor (2021). Additive effects ($a$) were sampled from a gamma distribution with scale and shape parameters equal to 1 and randomly assigned for each QTN. Similarly, dominance effects ($d$) for each QTN were computed by multiplying the absolute value of its additive effect (${a}_{i}$) by locus-specific dominance degree (${\delta }_{i}$​). Dominance degrees were sampled from a Gaussian distribution with ${\delta }_{i}\sim N\left({\mu }_{\delta },{\sigma }_{\delta }^{2}\right)$, where ${\mu }_{\delta }$ is the average dominance degree equal to 0.22 and ${\sigma }_{\delta }^{2}$ is the variance of the dominance degrees equal to 0.125. Therefore, at least a 26% chance that the delta will be negative (bidirectional dominance deviations) and a 1% chance that it will exceed the unit (overdominance).

The initial mean of the quantitative trait was 0, and its initial total genetic variance was 1. The phenotypic values of individuals were generated by adding the error, sampled from a normal (Gaussian) distribution, to the total genetic value of each individual. The initial values for broad-sense and narrow-sense heritability were set at 0.63 and 0.60, respectively, and these values were set according to the accuracy selection we have empirically observed in the LSU breeding program over the breeding stages (Table 1). Also, this study did not consider epistasis, although it may contribute to heterosis in rice (Huang et al. 2016). In this study, our primary objective was to assess genetic gain over time based on selection strategies. We therefore focused on genotype means per year or cycle, rather than simulating plot-level data in multi-environment trials. We acknowledge that including plot-level data and genotype-by-environment interactions would be important for more detailed modeling of trial efficiency or prediction accuracy, but it is not essential for the main goal of this manuscript, which is to evaluate the effectiveness of selection strategies in driving genetic gain.

**Table 1:** Empirically observed broad-sense (*H*^2^) and narrow-sense heritability (*h*^2^) for grain yield at each LSU rice breeding program breeding stage

Heritability	*F*_2_	*F*_3_	*F*_4_	*F*_5_	*F*_6_	*F*_7_
*h*^2^	0.03	0.15	0.40	0.60	0.70	0.80
*H*^2^	0.06	0.20	0.45	0.63	0.72	0.81

### Base population, burn-in phase, and the first GS training set

**Fig. 2:**
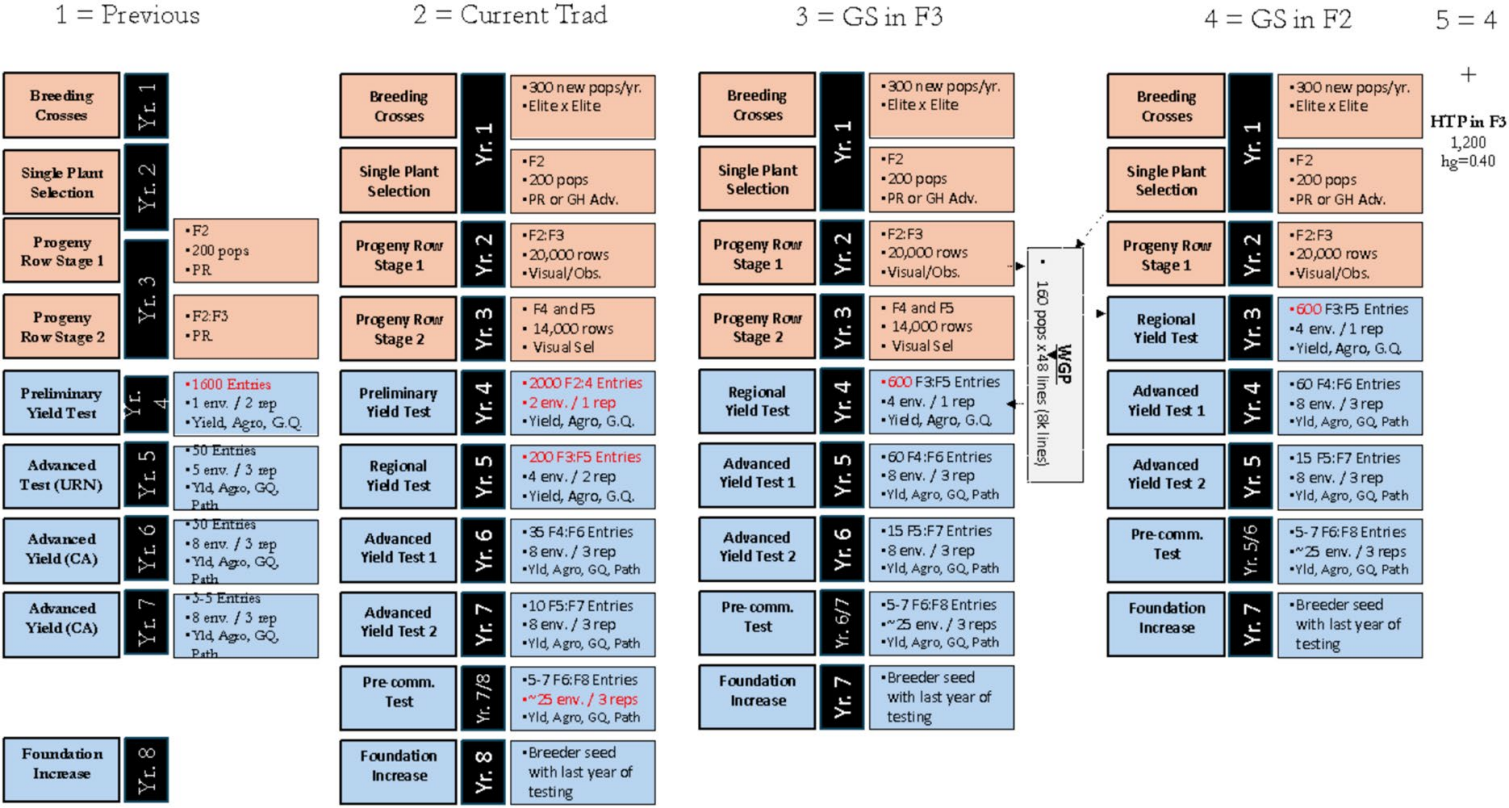
The five breeding frameworks used or to be tested in the LSU rice breeding program. The number of entries in red compose the most important breeding stages

The base population of 40 individuals was obtained from the initial 1000 lines of the historical population based on their superior phenotypic values. On average, the Ne was 28 when using 40 parents and roughly 70% of the parents in the other scenarios. We first considered a traditional rice breeding program named “Previous” (Fig. 2). The breeding scheme is an adaptation of the pedigree method. Thus, based on that, we simulated three selection cycles totaling 15 years of breeding in the burn-in stage. In each cycle, 40 parental lines were crossed to generate 160 F1 plants, which were selfed to produce 100 F2 plants from each cross. After three breeding cycles, we obtained the base population to evaluate the downstream scenarios of this study (Fig. 2).

Regarding the GS, the initial training set (TS) comprised 1152 inbred lines from 30 crosses between 60 individuals (parents), with nearly 40 plants per cross from the base population after the burn-in stage. The marker effects were predicted using the ridge-regression best linear unbiased prediction (RRBLUP) (Endelman 2011) according to the equation below:

$$y = 1\mu + Z_{u} u + \varepsilon$$

where $y$ is the vector of individual phenotypic values from the TS (adjusted means); $\mu$ is the mean (intercept); $u$ is the vector of marker effects, where $u \sim N\left(0,I{\sigma }_{u}^{2}\right)$; and $\varepsilon$ is the vector of random residuals. $1$ is the vector of ones and ${Z}_{u}$ is the incidence matrix of TS genotypes for $m$ markers. ${Z}_{u}$ is coded as 1 for homozygous *A*_1_*A*_1_, −1 for homozygous *A*_2_*A*_2_, and 0 for heterozygous A1A2.

To perform the GS, the genomic estimated breeding value (GEBV) was estimated using the following equation: $\text{GEBV}=\text{Mu}$, where $M$ is the incidence matrix of selection candidate genotypes, and $u$ is the vector of predicted marker effects.

### Comparing Breeding methods and the deployment of modern tools

First, we compared three different breeding frameworks used in the LSU rice breeding program in the last 40 years and two scenarios planned for the coming years (Fig. 2). The "Previous" method corresponds to the traditional phenotypic-based breeding scheme used for more than 30 years until 2017. The current "Current_Trad" was an adaptation of the "Previous,” including one more stage of phenotypic evaluation. It was used mainly for 5 years, and its importance has decreased yearly due to the deployment of genomic selection.

Regarding the “modern” era, the "GS in F3" corresponds to the application of genomic selection on the F_3_ generation and update of training population and markers effect on the last three breeding cycles, as described by Sabadin et al. (2022), based on the preliminary yield test (PYT) data, Then, to reduce the breeding cycle, we designed the “GS in F2” scheme breeding cycle with the application of GS on the *F*_2_ population. The TS and marker effects updates are done with the *F*_3:5_ population from the last three breeding cycles. Finally, the fifth scenario, "GS.F2_HTP.F3," corresponds to the fourth one, plus the deployment of high-throughput phenotyping (HTP) for grain yield on the F_3_ population. In this case, there will be another round of selection (1200 genotypes) at the progeny row stage under an estimated accuracy of 0.40 (empirical results not present here).

**Fig. 3:**
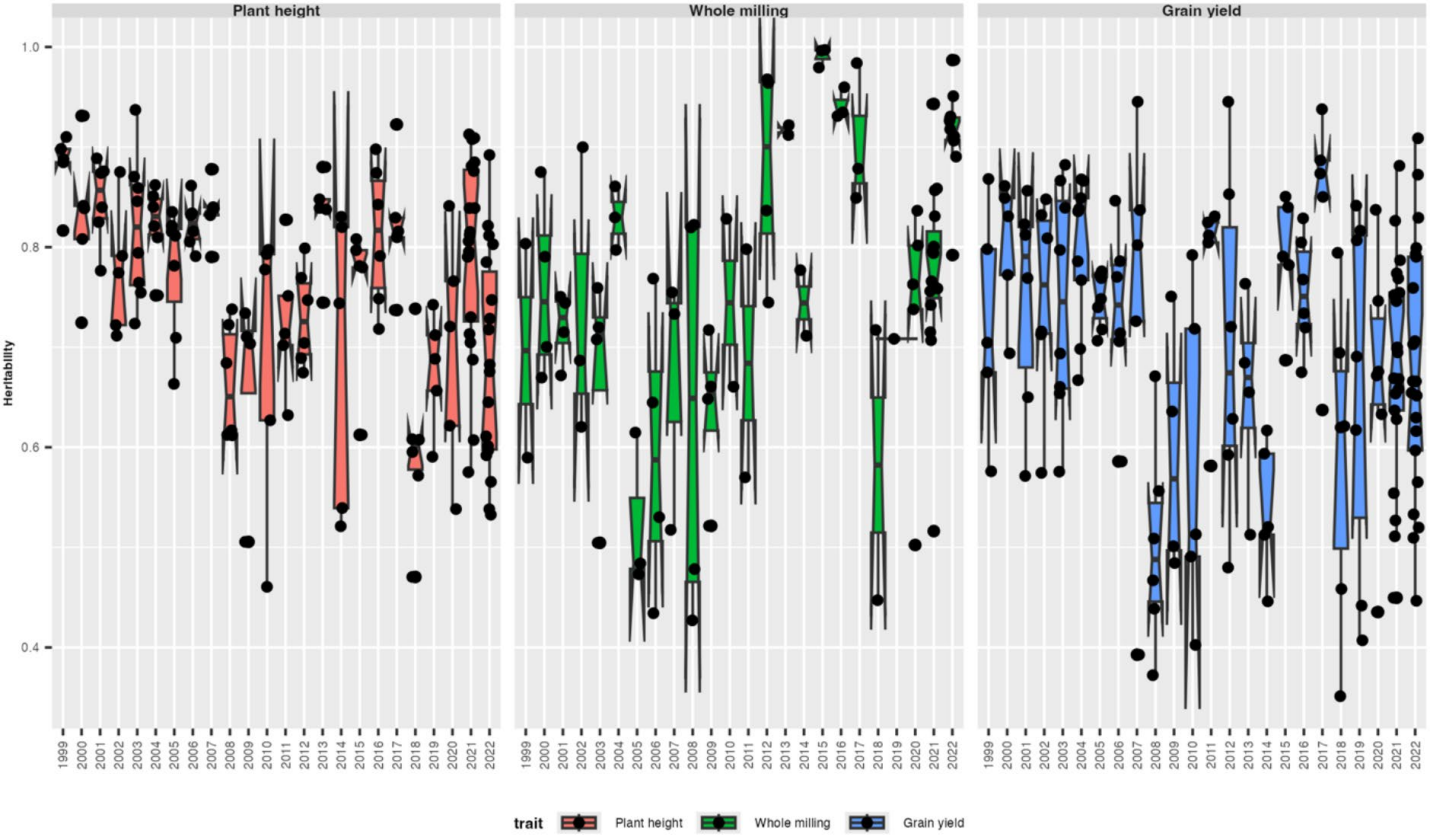
Heritabilities over the years

The 40 parents are selected from the PYT and the advanced yield test (AYT). Moreover, the number below corresponds to the whole breeding program, with five main market segments (Fig. 2). For the simulations, we adjusted the number to 80% of them, representing the long-grain efforts, which is the most important for our target market (Fig. 3).

For 50–60 years, the LSU rice breeding program relied mainly on introductions. Then, the more recent and intense hybridization era was considered a burn-in, matching Dr. Steve Lipscombe’s phase (21–24 years), representing three breeding cycles. Then, the optimization represents the Dr. Famoso era and the plans for the rice breeding framework, including modern tools and adjustments in the selection process.

### Number of parents, crosses, and siblings

Based on the best breeding framework scenario identified in the simulations described above ("GS.F2_HTP.F3″), we aimed to determine the best combination of the number of parents to be used in the crossing block (20, 40, 60, or 80), crosses (80, 12, 160, or 200), and progeny size (50, 100, 150, 200). Consequently, we compared 64 breeding number combinations. Each scenario was independently simulated over 100 replicates, totaling 6400 simulation runs (64 combinations × 100 replicates). Each replicate simulates an independent breeding program trajectory from the same burn-in population (pop.trad) but with stochastic variation in recombination, selection, and phenotypic expression. The use of 100 replicates per scenario was chosen to account for this stochasticity and provide robust estimates of central tendency and variance. To be more realistic with the practice, it is important to highlight that not all individuals were "genotyped” and went to GS. In this context, we considered a phenotypic negative selection of F_2_ plants; only 70% of the superior plants are advanced.

## Results

### Genetic trends in the last decades

The heritability calculated for plant height (the first box in red), whole milling (the second box in green), and plant yield (the third box in blue) showed high variance across the years for all the traits. Most values and central tendency across years are distributed above 0.6, and the distribution showed similar fluctuation patterns across the traits. The main difference is due to the variability of the values, where the heritabilities to plant height have presented less variation across years than the values of whole milling and grain yield.

**Fig. 4:**
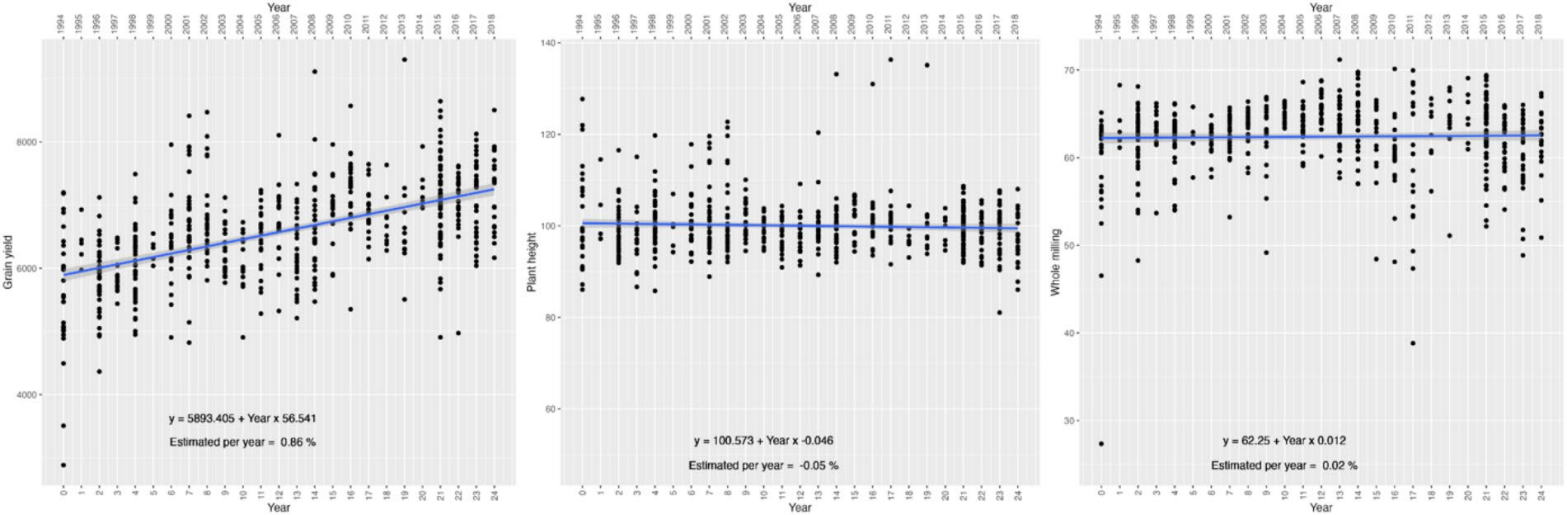
Plant height (centimeters; on the left), whole milling (percentage; in the center), and grain yield (kg/ha; on the right) performances for all the lines obtained from 1994 and 2018

The plant height showed a minimum value of 81.06 cm and a maximum of 136.3 cm. The linear regression of the plant height values against the year trial from 1994 to 2018 revealed a negative slope of -0.044, with an estimated reduction of plant height of around −0.04% per year. Despite the negative slope, we could observe that the values still have a good distribution around the mean of 100 centimeters across the years and a reduction of extreme values (Fig. 4—on the left).

We observed that the whole milling percentage values are concentrated at 60%, with a minimum value of 27.38% and a maximum value of 71.21%. The breeding program achieved a modest increase of approximately 0.02% per year in the whole milling percentage. The data dispersion did not change significantly over the years, indicating that this trait had a decent amount of variability (Fig. 4—in the center).

Finally, we observed a clear, constant, and positive trend for grain yield. Due to the genetic effect, grain yield increased by 0.86% per year, or approximately 56.54 kg/ha per year, for the lines obtained from 1994 to 2018. Besides the increase in productivity, we could maintain the data variation range across the years.

### The genetic gains

**Fig. 5:**
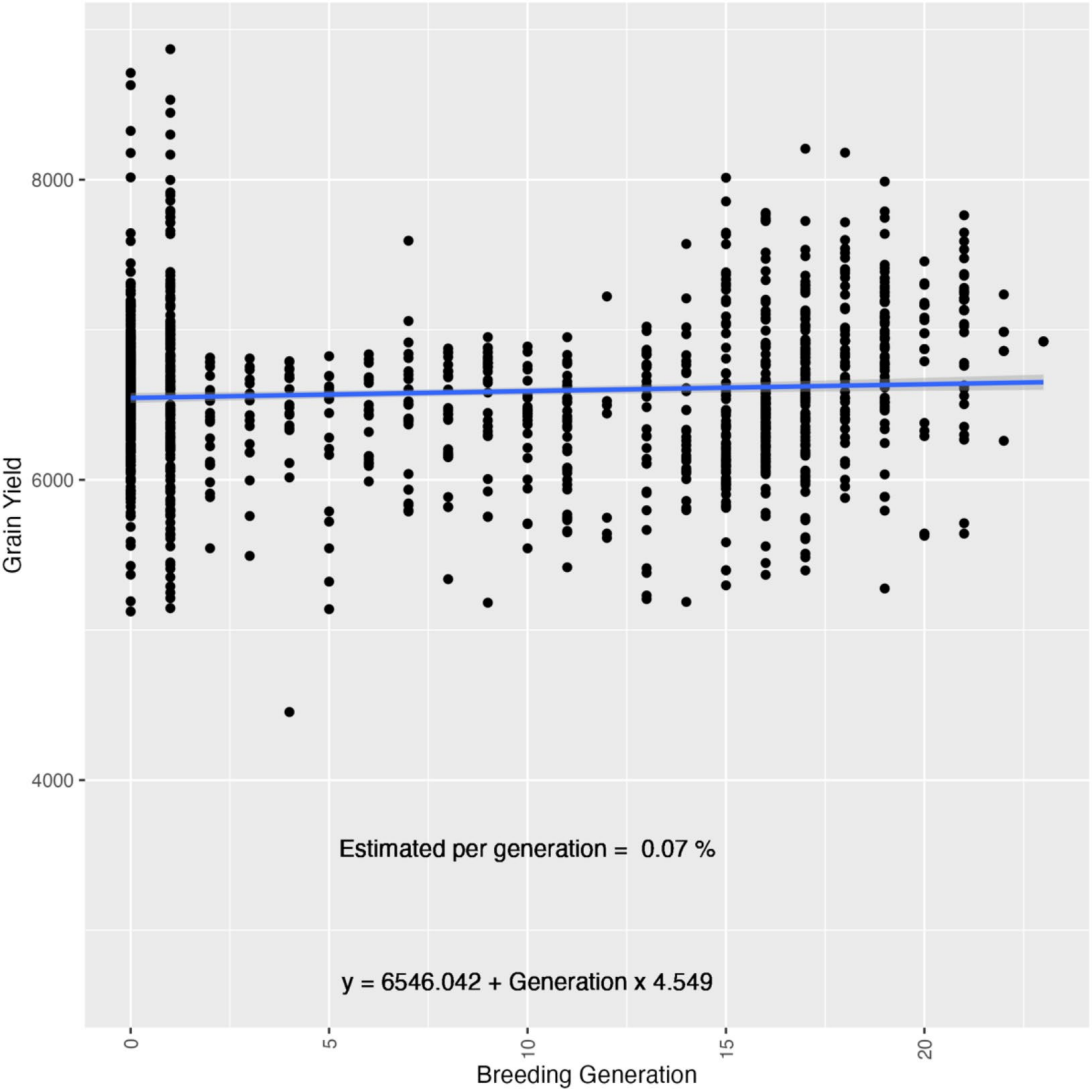
Additive genetic gains for grain yield (kg/ha) over 110 years (1908–2018) in the LSU rice breeding program, comprising 23 generations of selection

The regression analysis of the breeding values for yield across generations showed an estimated gain of 0.07%, which means a small but constant increase of approximately 4.55 kg/ha per generation over 23 generations of the rice breeding program spanning 110 years (Fig. 5). We also observed significant variability in the trait across the breeding generations. This steady improvement underscores the efficacy of the breeding program in enhancing grain yield through successive generations. Despite the overall positive trend, significant variability was observed in the trait across different breeding generations. This variability highlights the influence of both genetic and environmental factors on the breeding outcomes. The scatter plot shows the distribution of grain yield across generations, with the regression line illustrating the average trend of genetic gain over time.

**Fig. 6:**
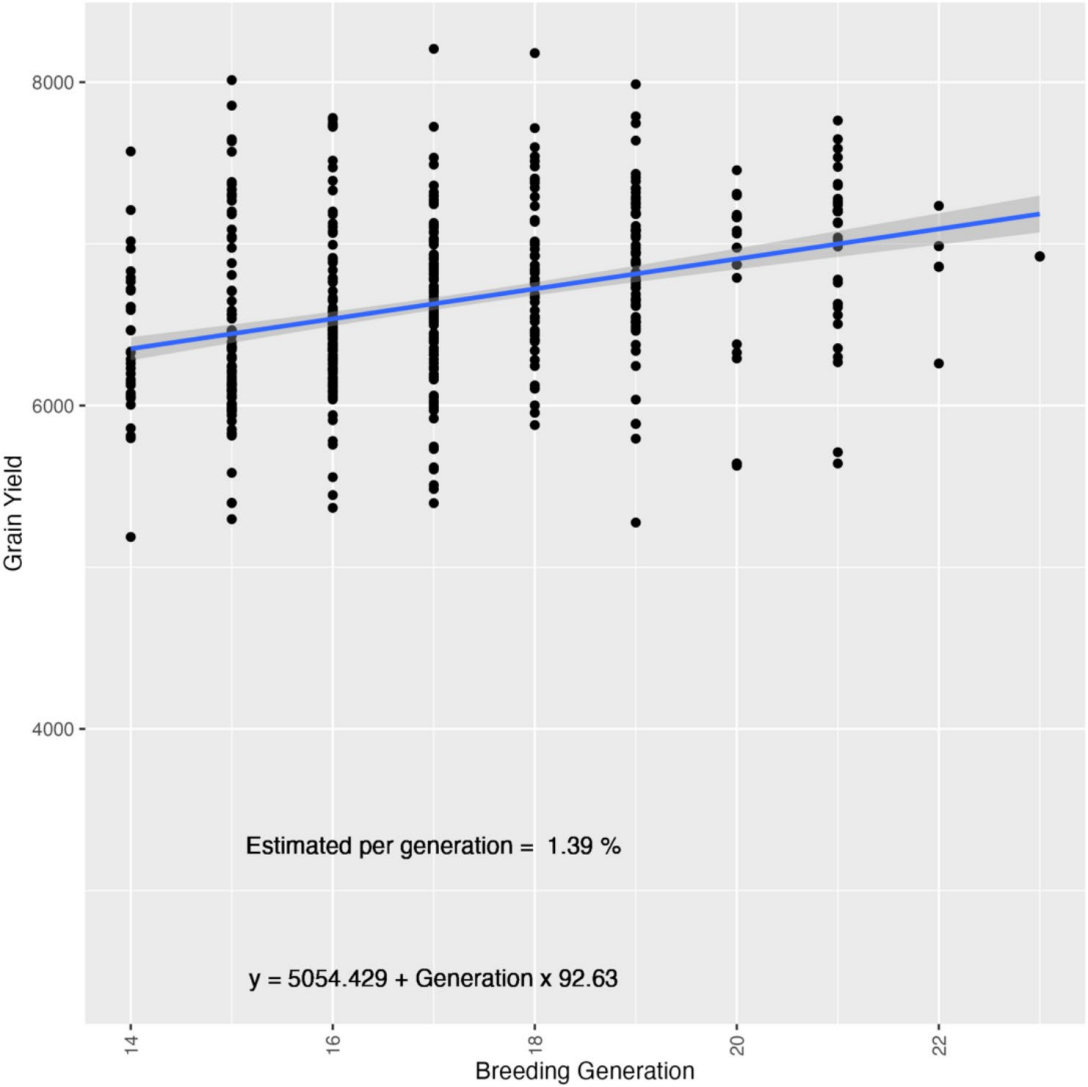
Additive genetic gains for grain yield (kg/ha) over 50 years (1968–2018) in the LSU rice breeding program, comprising 10 generations of hybridization and selection

We noticed a more pronounced trend in the genetic gains during the last 10 breeding generations, leading us to conduct a detailed analysis for this interval. This key transition represents a shift in the breeding program from germplasm introduction and evaluation to hybridization and recycling of parents. When we examined only the last 10 generations, encompassing 50 years, we found a greater genetic gain per generation of 1.39%, reflecting an increase of 92.63 kg/ha per generation or 18.43 kg/ha per year (Fig. 6).

### Comparing breeding frameworks via simulations

**Fig. 7:**
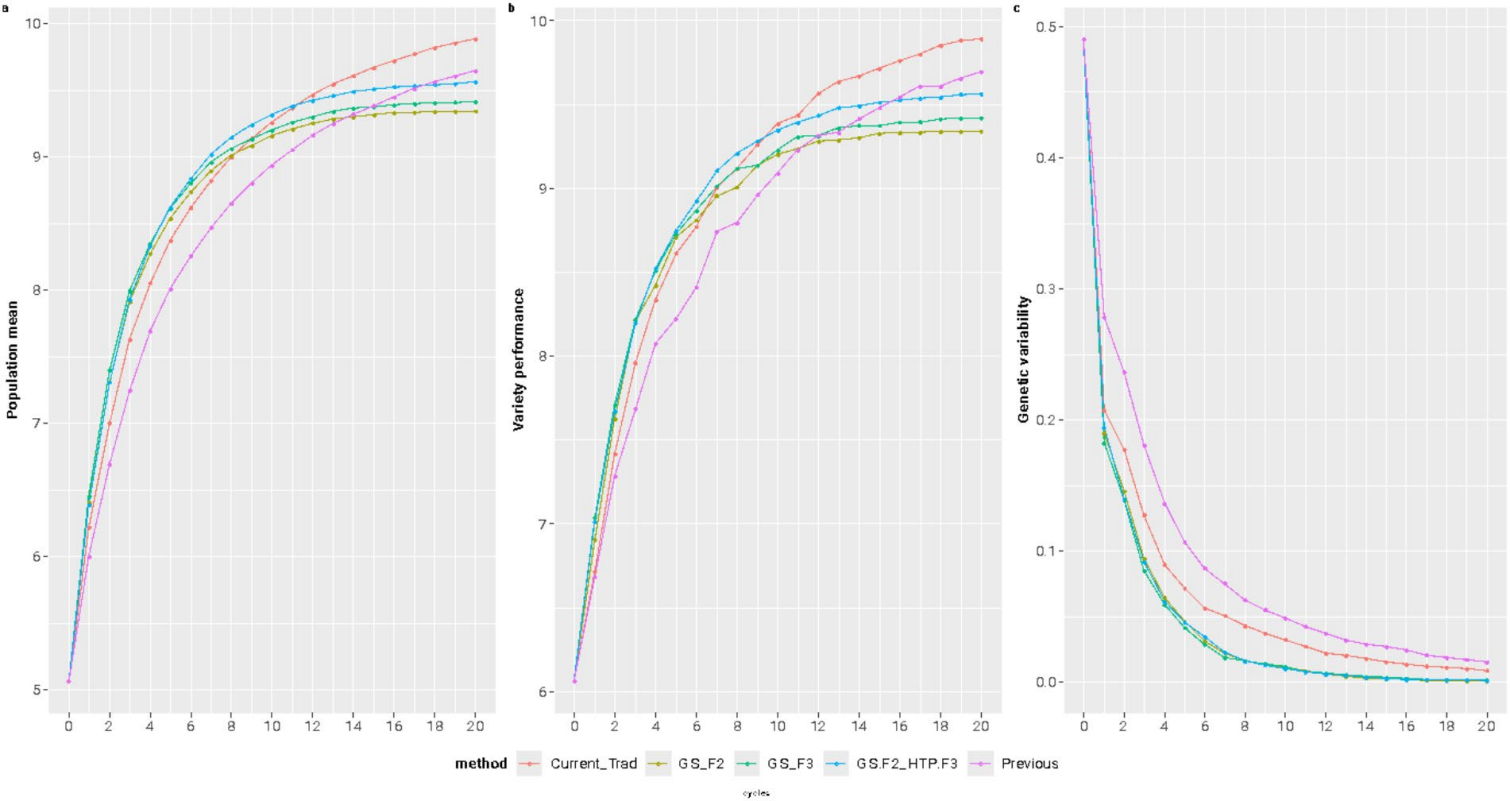
The average population performance (**a**), the best line (potential variety) performance (**b**), and the genetic variability (**c**) for each one of the five breeding methods over 20 breeding generations

**Fig. 8:**
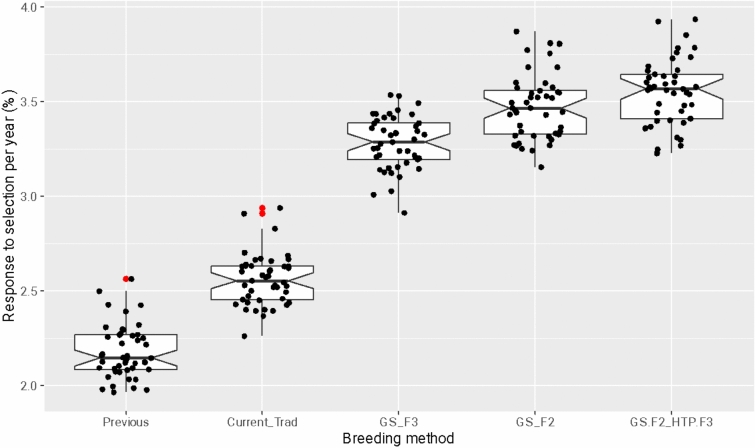
The average gains per year (%) in terms of population mean for each of the five breeding frameworks over 15 years of breeding. The red dots represent outliers observed in the simulations

Regarding population performance, we see that almost all the selection methods reached a plateau between 10 and 14 cycles; however, they did so at different levels. Besides the close values, we noticed that the "Current_Trad" method performed best at the 20th cycle, followed by “Previous” and the GS-based methods. In the first breeding cycles, the best variety performance followed the same pattern as the average population performance in almost all the methods (Fig. 7a, b). Concerning genetic variability, the phenotypic-based selection methods preserved more than the GS-based methods (Fig. 7c). Besides being interesting, these results do not provide a fair comparison because each breeding framework has a different length (Fig. 2). Therefore, we set a horizon of 15 years of breeding and estimate the genetics gain per year (%) for each scenario tested (Fig. 8).

In this case, the "Previous" method exhibited a moderate gain of 2.2% annually. The "Current_Trad" method demonstrated an improved performance with an increase of 2.6% annually. The "GS_F3" method, utilizing genomic selection, further enhanced the response to 3.3% per year. The "GS_F2" method showed another increase, achieving a 3.45% annual gain. Finally, the highest response was observed in the "GS.F2_HTP.F3" method, which combined genomic selection with high-throughput phenotyping, yielding a remarkable 3.65% gain per year. These results underscore the superior efficiency of advanced genomic and phenotyping techniques in accelerating genetic gains in plant breeding.

### The balance between the number of parents, crosses, and progeny size

**Fig. 9:**
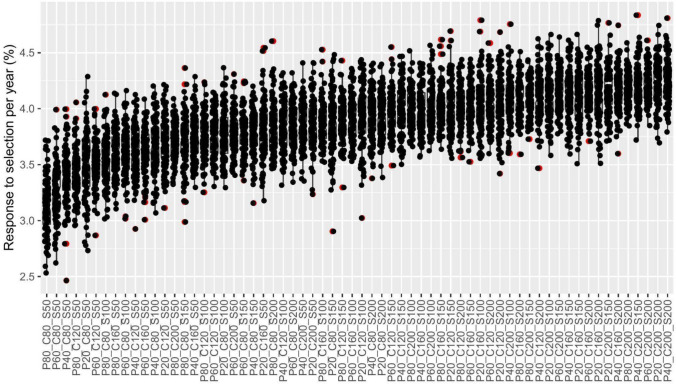
Response to selection over 15 years of breeding with different combinations of the number of parents (P), number of crosses (C), and progeny size (S). The red dots represent outliers observed in the simulations

Our results demonstrate that even the best breeding framework (“GS.F2_HTP.F3”) can be further improved by finding the best combination of numbers of parents, crosses, and progeny sizes (Fig. 9). The response to selection per year ranged from approximately 2.5–4.5% between combinations. Overall, combinations with more crosses (C) and mainly larger progeny sizes (S) showed better responses to selection per year (Fig. 9 and Table 3).

Furthermore, the analysis of variance (Table 2) highlighted the significance of the factors of the number of parents (P), number of crosses (C), and the progeny size (S) on the response to selection. Also, the importance of each one of these components (VC%) in explaining the improvements in population performance. In this context, these results of the variance component showed that the progeny size had the major effect, accounting for around 35.9% of the total variance, followed by the number of crosses with 23%. The number of parents had the lowest impact on the results and explained 3.3% of the total variance (Table 3).

**Table 2:** Analysis of variance for the factors P (number of parents), C (number of crosses), S (progeny size), and their interactions

Source	Df	SS	MS	*F* value	Pr(> *F*)	VC%
P	3	21.706	7.235	166.6439	< 2.2e−16***	3.36
C	3	136.140	45.380	1045.2125	< 2.2e−16***	23.01
S	3	212.693	70.899	1632.9415	< 2.2e−16***	35.92
P:C	9	1.725	0.192	4.4141	8.887e−06***	0.31
P:S	9	4.884	0.543	12.4998	< 2.2e−16***	1.03
C:S	9	3.569	0.397	9.1331	7.373e−14***	0.73
P:C:S	27	1.140	0.042	0.9726	0.5044	0.00
Residuals	6336	275.091	0.043			35.65

Degrees of freedom (Df), the sum of squares (SS), mean squares (MS), *F* values, and *p*-values (Pr(> *F*)), and the percentage of the total variance (VC%) explained for each source in the model

Significance codes: 0 ‘***’ 0.001 ‘**’ 0.01 ‘*’ 0.05 ‘.’ 0.1 ‘ ’ 1

**Table 3:** The first three groups of population performances and 16 best combinations of the number of parents (P), number of crosses (C), and progeny size (S), obtained via the Scott–Knott test

P_C_S	Population performance	G1	G2	G3
P40_C200_S200	9.6	a		
P20_C200_S200	9.56	a		
P60_C200_S200	9.54	a		
P40_C200_S150	9.54	a		
P80_C200_S200	9.53	a		
P40_C160_S200	9.51		b	
P20_C200_S150	9.5		b	
P20_C160_S200	9.48		b	
P60_C160_S200	9.48		b	
P20_C160_S150	9.47		b	
P40_C160_S150	9.47		b	
P60_C200_S150	9.47		b	
P20_C200_S100	9.45			c
P40_C120_S200	9.44			c
P80_C200_S150	9.44			c
P80_C160_S200	9.43			c

^(^^1^^)^Means followed by equal letters in the columns belong to the same grouping, according to Scott–Knott's test, at 5% probability

Finally, to identify which combinations are significantly different in response to selection, we compared the scenarios via the Scott–Knott test, which effectively grouped all breeding strategies into distinct categories. The Scott–Knott test was chosen specifically because it organizes treatments into disjoint groups based on statistical significance, simplifying the identification of clusters of superior combinations without the complexity of interpreting numerous pairwise comparisons. This approach helped us determine an optimal balance between high performance and practical affordability. Combinations with higher numbers of crosses and larger progeny sizes tend to perform better, as seen in Group 1 (Table 3).

## Discussion

### Heritability and genetic connectivity

Regarding heritability estimates, most of the values and central tendency across years are distributed above 0.6 values, indicating a strong genetic component and reliable data. This pattern was observed for plant height, whole milling, and yield. Heritability values for plant height fluctuate less over time than whole milling and yield. This suggests a more stable genetic control for plant height, attributed to fewer environmental interactions or more efficient selection practices (Yan et al. 2023). In contrast, the heritabilities of whole milling and yield display greater variability. This is because yield and whole milling are more quantitative traits; they are influenced by multiple genes and environmental factors, leading to complex interactions that affect their heritability (Saeidnia et al. 2023).

Various factors can introduce errors in genetic gain estimates, including breeding scheme, trait heritability, connectivity, and the number of trials and sites per variety (Rutkoski 2019a, b; Raymond et al. 2023). Therefore, direct estimates of genetic gain should not be used to compare breeding programs. Instead, genetic gain estimates should be employed to assess the presence of a positive upward trend or to obtain a close estimate of the realized genetic gain within a breeding program (Rutkoski 2019a, b). Breeding programs have a major impact on increasing crop yield at the necessary rate to match the needs of the world's growing population and achieve global food security (Cobb et al. 2019). One of the strategies to track the performance of a breeding program on this mission is the estimation of the genetic gain (Xu et al. 2017).

Therefore, genetic connectivity is critical to estimating genetic gain in a given period based on historical data. The lack of connectivity can lead to biased estimations by confounding genetic and year effects (Rutkoski 2019a, b). The connectivity values present in our study ranged from 17 genotypes (2003–2004) to 43 genotypes (2016–2017) shared genotypes between sequenced years. Consequently, the data have a considerable degree of connectivity over the years, which is desirable for reliable estimates of genetic gain. Our connectivity is similar to historical studies that estimated genetic gain on yield, such as those by Rahman et al. (2023), which had connectivity ranging from 16 to 45 genotypes between years, and Khanna et al. (2024), with connectivity ranging from 6 to 64 genotypes. To get low errors and a high correlation between the real and the estimated breeding values, studies of genetic gain with historical data also require a good long-term pedigree record within the breeding program to allow a good estimate of the additive relationship between the lines (Rutkoski 2019a, b). The main limitations of historical studies, connectivity, and pedigree records were overcome in the present study, which adds reliability to our results.

### Genetic trends in the last decades

Plant height is a critical factor in rice cultivation, as it is directly associated with lodging and grain yield (Wu et al. 2022; Seck et al. 2023). Rice breeders must find a balance between reducing plant height to minimize lodging and promote tillering while avoiding excessive reduction in height that could lead to smaller grain size and excessive tillering, ultimately reducing yield (Liu et al. 2018). With a minimal negative trend of -0.04%, our breeding program has successfully maintained plant height at a balanced length of around 100 centimeters, preserving trait variability and reducing outlier values over the years. Field management practices and environmental changes significantly influence rice plant height (Liu et al. 2018; Wu et al. 2022). Maintaining variability within our breeding program provides a buffer to adapt our selection process to these changes, mitigating potential yield losses.

Rice milling is a quantitative trait due to several QTLs and many instances of epistasis (You et al. 2022; Ali et al. 2023). Despite the modest positive genetic trend of 0.02% per year, the breeding program could maintain constant improvement for milling with the predominance of milling rates above 60%. Moreover, it has a high genotype × environment effect, and this interaction makes it challenging to achieve consistent genetic improvement in milling across different environments or/and years (Cruz et al. 2021; Ali et al. 2023). Cruz et al. 2021 observed a negative additive genetic trend of −0.16% in the Latin American Fund for Irrigated Rice breeding program over 20 years of selection. They highlighted the complexity of improving the trait and how applying MAS and GS, which consider genotype × environment interactions, can enhance the rate of genetic gain for milling.

To match food and biofuel demands to meet the projected human population in 2050, the so-called “2050 challenge,” we will need an increase in crop yield at the annual rate of 2% (Li et al. 2018). We observed a positive trend with a rise of 0.86% per year due to the additive genetic effect, which increased to approximately 56.54 kg/ha per year from 1994 to 2018. During this period of 24 years, we could bring the mean value for grain yield from 5893 to 7250 kg/ha, which means an increase of 23%. Despite the significant increase that we have made, we are aware that we need to improve our genetic grain rate and increase our selection efficiency with the adoption of new strategies and breeding tools, such as genomic selection, which is a reality in our breeding pipeline. Besides the main objective of increasing yield, a sustainable breeding program should effectively utilize genetic diversity to achieve genetic improvement in specific traits while preserving genetic diversity in non-target loci (Meuwissen et al. 2020). Maintaining genetic diversity within a breeding population presents a significant challenge for achieving long-term genetic gains. We could observe the presence of great variability in the distribution of values for the three traits evaluated (plant height, whole milling, and grain yield).

### Genetic gain in 110 years

The population breeding value improved over 110 years and 23 breeding generations by approximately 0.07% per generation, equating to an average increase of 4.55 kg/ha (Fig. 5). This steady improvement underscores the efficacy of the breeding program in enhancing grain yield through successive generations. Despite the overall positive trend, significant variability in grain yield was observed across different breeding generations, highlighting the influence of genetic and environmental factors on grain yield. A more pronounced genetic gain was observed during the last 10 generations of the breeding program. During this period, encompassing 50 years, the genetic gain per generation increased to 1.39%, reflecting an average increase of 92.63 kg/ha per generation. Rice cultivation in the USA has been done since the 1600s using cultivar introductions (Wang et al. 2024). In the early 20th century, advancements in understanding inheritance laws led to rapid progress in genetic disciplines and applied plant breeding. In this period, the US rice experiment stations began implementing directed hybridization and artificial cross-pollination with rice materials to generate genetic variability and then, the selection of adapted materials to the Southern US conditions aiming for stability and higher yields (McKenzie et al. 2015; Wand et al. 2024). This marked improvement in the last 10 generations is attributed to a shift in breeding strategies from introducing genotypes from other regions to performing hybridization between stable materials within the programs (Fig. 6).

The observed genetic gains in grain yield through successive generations of selection indicate the long-term effectiveness of the breeding program. The greater genetic gain in the most recent generations suggests that modern breeding techniques, including hybridization and the selection of stable genotypes, have contributed significantly to this improvement. Future breeding efforts could benefit from continuously integrating established modern tools such as GS techniques, HTP, and optimized statistical models to enhance further the accuracy and efficiency of selecting superior genotypes. Studies have shown that incorporating genomic selection can yield substantial genetic gains by accelerating the breeding cycle and improving selection precision (Biswas et al. 2023). HTP tools can increase selection accuracy at lower costs and with higher selection intensity, enabling the phenotyping of more plots (Xu et al. 2021). The combination of GS and HTP has great potential to be the next turning point in the breeding program based on the results of our simulations.

## Optimizing for the future

Concerning the trend of genetic variability, the methods based just on phenotypic selection had a slow rate of variability reduction compared to methods that use genomic information. Marker-based BLUP methods are naturally based on the covariance between genotypes. They will cause relatives to be co-selected more frequently, reducing genetic variability faster than phenotypic selection (Heslot et al. 2015). Fritsche-Neto et al. (2024) also observed this trend in a study that simulated a rice breeding program. The genomic selection methods showed a greater reduction in genetic variability than traditional phenotype-based methods (Fritsche-Neto et al. 2024). This pattern was also observed in other studies that simulated the use of genomic selection in various breeding programs across different systems, including sorghum (Muleta et al. 2019), maize (DoVale et al. 2022), pulses (Li et al. 2022), trees (Degen and Müller 2023), livestock (Wientjes et al. 2022), and chickens (Pocrnic et al. 2023). Our results also suggest that the reduction of genetic diversity was inversely proportional to the genetic gain and the variety performance. The methods that implemented GS had an accelerated rate of genetic gain and variety performance in the initial cycles, and this was reflected proportionally in the genetic diversity loss. The final results at the 20th cycle also showed the inverse proportion; methods based on phenotypic selection could preserve more genetic variability and presented higher final values for population mean and variety performance compared with methods with GS.

The response to selection highlighted the potential of implementing GS when considering the possibility of shortening the breeding cycle. After simulating 15 years of a breeding program to compare all the methods with their correspondent breeding cycle length, we had three breeding cycles of "Previous and the "Current_Trad," 4 of the "GS_F3," and 5 of the "GS_F2" and "GS.F2_HTP.F3." The "Previous" method shows a moderate response to selection even when compared to the “Current_Trad.” This suggests substantial increases in response to selection can be obtained by including another round of phenotypic selection, increasing the overall framework accuracy without penalizing the cycle length.

Regarding genetic gains observed via simulations, usually, they are typically overestimated. Therefore, our objective is not to focus on the actual numbers but to compare breeding frameworks and determine which will provide a relatively better genetic gain at a lower cost. In this context, all the GS methods outperformed the methods based on phenotypic selection, even when the methods had almost the same breeding cycle length. The selection based on estimated breeding values improves the probability of selecting the top-performing lines, increasing the selection differential and the genetic gain (Cobb et al. 2019). Another main advantage of GS is reducing the breeding cycle length while enhancing the expected genetic gain and selection response per unit of time (Crossa et al. 2017). The reduction of the cycle length significantly impacted the response to selection between the methods. However, from our perspective, the key distinction between the phenotypic-based and GS-based methods is due to the enhanced accuracy in selecting the top-performing lines in the early stages (Heslot et al. 2015; Hickey et al. 2017). Furthermore, the “GS.F2_HTP.F3” forecasts the implementation of GS on the *F*_2_ generation and HTP in *F*_3_, resulting in the highest response to selection per year, mainly because it replaces a low accurate phenotypic selection in those stages for a better and more precise strategy and of course, with the lowest cost.

Regarding the optimization of breeding numbers, our results have practical implications for rice breeding programs without abrupt changes in logistics or substantial costs. It is well-known that the maximization of genetic gain and the maintenance of genetic variability are achieved with the increase of the number of parents to ensure a wide genetic base, the conduction of more crosses to generate diverse progeny, and the utilization of larger progeny sizes to enhance selection accuracy and genetic gains. Fritsche-Neto et al. (2024) also observed the same results in a simulation study with rice, where a bigger population size generated higher genetic gain and better maintenance of genetic variability over a small population size. The response to selection per year varied between approximately 2.5–4.5%, with higher numbers of crosses and larger progeny sizes showing better responses (Fig. 9). Furthermore, the variance component analysis (Table 2) revealed that progeny size had the major effect on the total variance, contributing 35.92%, followed by the number of crosses (23.01%) and the number of parents (3.36%). This hierarchy of influence underscores the critical role of progeny size in breeding programs, as larger progeny sizes ensure a broader genetic base, facilitating the selection of superior individuals. Consequently, this result may help define the best strategies in resource allocation and shed light on the old question: Lees and big or more and smaller progenies?

In this context, a smaller population size will maximize the rate of allele fixation and boost the reduction of genetic variability; smaller populations will also influence genetic drift (Fritsche-Neto et al. 2024). Maximizing the population size not only mitigates the negative effects of genomic selection and drift over the genetic variability and allele fixation, but it will also increase the genetic gain with the possibility of higher selection intensity (Xu et al. 2017), and the Mendelian sampling, hardening the genomic selection advantages. However, increasing the population size of a breeding program is not simple. Although the main objective is to constantly increase genetic gain, a breeding program must also be cost-effective to keep its sustainability. Using GS can make it possible to test more lines, increasing the selection intensity and genetic gain. Still, there is a trade-off between the accuracy generated by the markers and the genotyping costs.

The allocation of resources regarding the number of parents, crosses, and progeny size will depend on the breeder's interest in maximizing the variation among or within families. It will also depend on factors such as family correlation and the number of traits to evaluate (Covarrubias-Pazaran et al. 2021). Hence, finding an equilibrium between those numbers and the cost to genotype is key to optimizing the cost-benefits of genomic selection within a breeding program. In this context, simulation studies can help us to test a wide range of conditions within a breeding program under a certain budget, designing the best strategies to maximize genetic gain per amount invested (Muleta et al. 2019; Li et al. 2022).

## Conclusion

The results of this study demonstrate that the Louisiana State University (LSU) rice breeding program has achieved substantial genetic gains over 110 years, with a notable increase in grain yield. These gains were most pronounced over the last 25 years, and recent modifications have been simulated to increase the rate of gain. Also, methods based on genomic selection stood out for their superior selection response when compared to phenotypic methods. Simulations demonstrate that additional gains can be realized by modifying the existing program and incorporating new approaches, such as HTP. Therefore, the breeding method, which combines genomic selection (GS) and high-throughput phenotyping (HTP), has proved highly effective and is a priority for the coming years. Moreover, a better combination of the number of parents, crosses, and progeny sizes will be placed, not the best tested, but the best we can afford (the 4th). Finally, our results reinforced the importance of modern selection tools and strategic resource allocation to optimize program efficiency. They underscored the necessity of continuously adopting new technologies and breeding strategies to ensure the sustainability of breeding programs and meet future global food demands.

## Data Availability Statement

The datasets generated during and analyzed during the current study are available in the Mendeley Data repository [https://data.mendeley.com/datasets/67m9hwhrbg/1], with the 10.17632/67m9hwhrbg.1

## Supplementary Information

Below is the link to the electronic supplementary material.

Supplementary file1 (DOCX 598 kb)

Supplementary file2 (CSV 18 kb)
