# Supplementary material for: 110 years of rice breeding at LSU: realized genetic gains and future optimization
Source: Theor Appl Genet. 2025 Jun 9;138(7):142. doi: 10.1007/s00122-025-04913-z (PMC12149018; doi:10.1007/s00122-025-04913-z)
Supplement: Supplementary file 1 — Supplementary file1 (DOCX 598 kb) [file 122_2025_4913_MOESM1_ESM.docx]

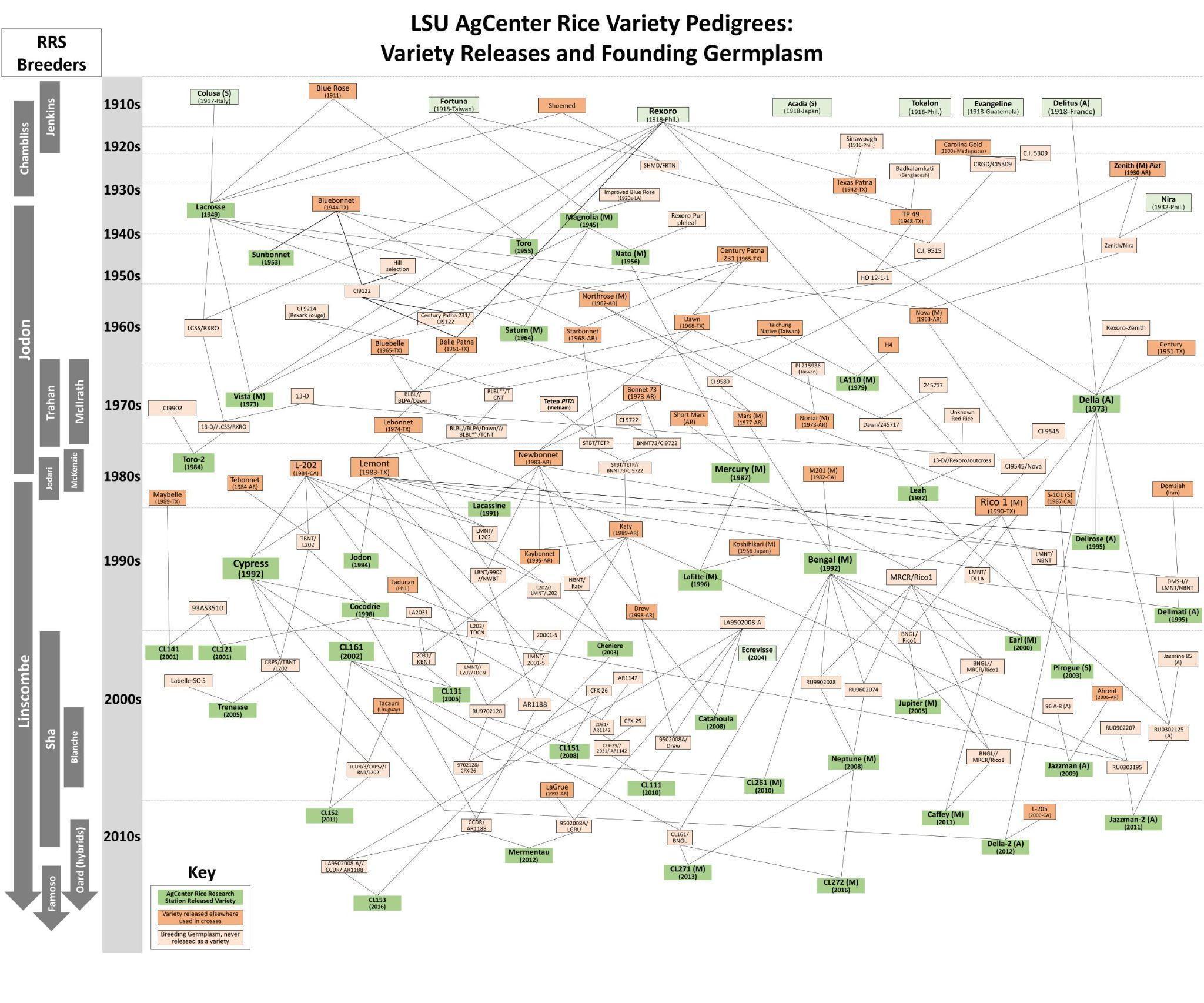


**Supplementary Figure 1.**  An overview of the LSU Rice breeding program’s historical pedigree, from the founders to the present

**

**

**Supplementary Figure 2.**  Genetic correlation among the evaluated traits.


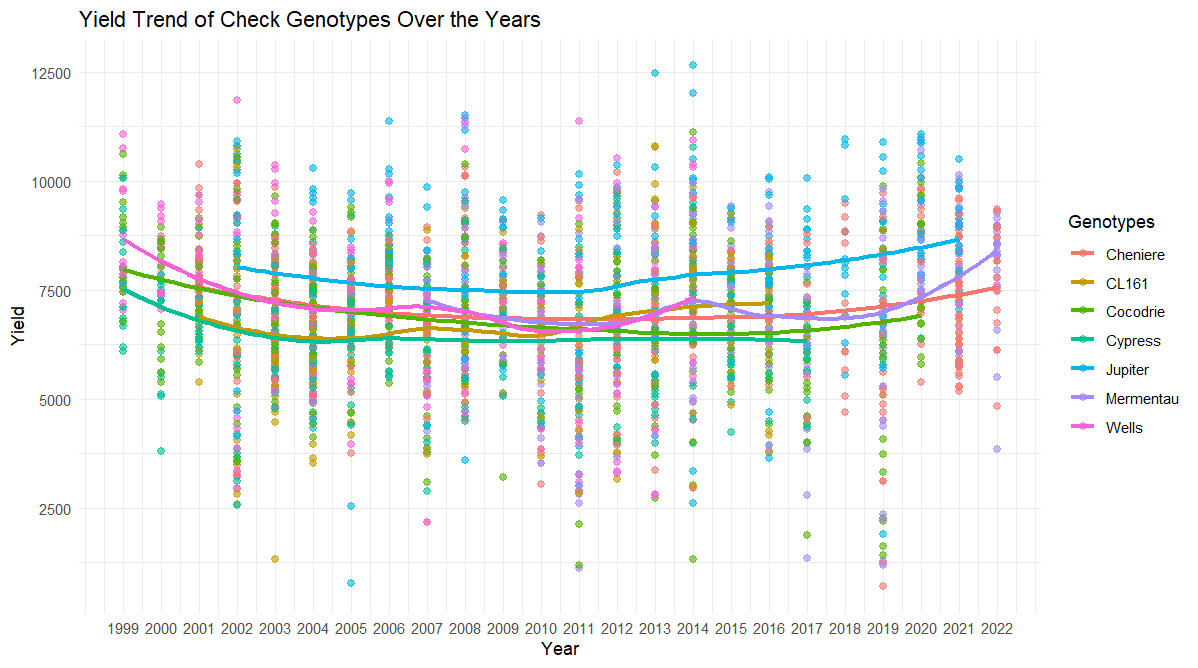


Supplementary Figure 3. Yield trend of the checks over the years calculated with checks present in at least 60% of the years.
